# Laparoscopic - assisted percutaneous nephrolithotomy as an alternative in the treatment of complex renal calculi in patients with retrorenal colon

**DOI:** 10.1590/S1677-5538.IBJU.2017.0043

**Published:** 2018

**Authors:** Petronio Augusto de Souza Melo, Fabio Carvalho Vicentini, David Jacques Cohen, Marcelo Hisano, Claudio Bovolenta Murta, Joaquim Francisco de Almeida Claro

**Affiliations:** 1 Divisão de Urologia, Centro de Referência da Saúde do Homem, Hospital Brigadeiro, São Paulo, SP, Brasil

## INTRODUCTION

Our aim is to demonstrate our technique and describe our experience using laparoscopic-assisted percutaneous nephrolithotomy (LA-PNL) as an alternative in the treatment of large renal calculi when retrorenal colon turns conventional PNL into a high-risk procedure.

## MATERIALS AND METHODS

We reviewed our prospective database of PNL in the last 5 years and identified all cases that underwent to LA-PNL. The option for choosing LA-PNL was made by the surgeon on the preoperative consultation based on the colon position on the CT scan. A 3-port laparoscopy was done for colon retrieval. Then, a percutaneous access guided by retrograde pyelography was performed, with direct visualization of the needle and dilators entrance. An ultrasonic device was used for stone fragmentation. A flexible nephroscopy was done at the end of the surgery. A nephrostomy tube was left in place and the trocars were removed.

## RESULTS

Nine patients were included in this series. Mean age of patients was 35.2 years. Mean stone largest diameter was 3.7cm. Eight patients were classified as Guy's Stone Score (GSS) 3 in regard of complexity and one patient was considered as GSS 4. Mean operative time was 152.2 min. A stone-free status was achieved in four patients and residual stones were seen in five patients. Mean hospitalization time was 2.41 days. No patient had any kind of peri or post-operative complications.

## CONCLUSIONS

Laparoscopic-assisted percutaneous nephrolithotomy is a safe procedure that can be considered when facing complex cases associated with retrorenal colon.

## ARTICLE INFO

**Available at: http://www.intbrazjurol.com.br/video-section/20170043_Melo_et_al**

**Int Braz J Urol. 2017; 43 (Video #5): 405-6**

